# Apoyo social y autocuidado en pacientes con Tuberculosis Pulmonar Hospital Lima Este, 2020, Perú*

**DOI:** 10.15649/cuidarte.2083

**Published:** 2022-10-16

**Authors:** Mayela Cajachagua Castro, Janett Chávez Sosa, Aileen Chilón Huamán, Angela Camposano Ninahuanca

**Affiliations:** 1 Universidad Peruana Unión, Perú. E-mail: mayela@upeu.edu.pe Universidad Peruana Unión Universidad Peruana Unión Peru mayela@upeu.edu.pe; 2 Universidad Peruana Unión, Perú. E-mail: viki16@upeu.edu.pe Universidad Peruana Unión Universidad Peruana Unión Peru viki16@upeu.edu.pe; 3 Universidad Peruana Unión, Perú. E-mail: aileenchilon@upue.edu.pe Universidad Peruana Unión Universidad Peruana Unión Peru aileenchilon@upue.edu.pe; 4 Universidad Peruana Unión, Perú. E-mail: angelacamposano@upeu.edu.pe Universidad Peruana Unión Universidad Peruana Unión Peru angelacamposano@upeu.edu.pe

**Keywords:** Apoyo Social, Autocuidado, Tuberculosis Pulmonar, Enfermería, Social Support, Self Care, Tuberculosis Pulmonary, Nursing, Apoio Social, Autocuidado, Tuberculose Pulmonar, Enfermagem

## Abstract

**Introducción:**

Uno de los problemas de salud pública en Perú es la tuberculosis pulmonar, conocer la realidad desde diferentes ópticas permitirá el abordaje apropiado para la terapéutica, así como la atención a la persona**.**

**Objetivo:**

De terminar la relación que existe entre el apoyo social y el autocuidado de los pacientes de un hospital de Lima - Este, Perú.

**Materiales y métodos:**

Estudio con enfoque cuantitativo, correlacional y transversal. La población fue de 114 pacientes pertenecientes al PNCT. Se utilizó el muestreo no probabilístico por intención, aplicando los criterios de inclusión y exclusión, se obtuvo la muestra constituída por 100 pacientes. Se aplicó el Test MOS de Apoyo Social y el Test de Autocuidado. En la recolección de datos se tomó en cuenta las considera ciones éticas. Los datos fueron procesados en el software SPSS-24, y analizados con Estadística descriptiva utilizando frecuencias y porcentajes. Para el análisis inferencial se utilizó Chi cuadrado.

**Resultados:**

El sexo, la edad, el grado de instrucción y el estado civil, no tienen relación con el autocuidado de los pa cientes con TB con p-valor >0,05. El 69,2% de los pacientes que recibía esque ma de tratamiento para TB MDR presentaron un autocuidado inadecuado. El 100% de los pacientes que recibía tratamiento para TB sensible presentaron un autocuidado adecuado, con relación significativa con un p-valor de 0,000. El 83,3% de los pacientes que percibieron un apoyo adecuado presentaron un autocuidado adecuado, el 76,9% que percibió un apoyo escaso, calificó su au- tocuidado como inadecuado con relación significativa con un p-valor de 0,000. Resultados similares se observan para las dimensiones del apoyo social con un p-valor <0,05.

**Conclusión:**

Los pacientes se caracterizan por ser jóvenes, varo nes, solteros y recibir tratamiento para TB sensible. Existe relación significativa entre un adecuado apoyo social y un adecuado autocuidado. Un esquema de tratamiento para TB resistente se relaciona con un inadecuado autocuidado.

## Abstract (en)

**Introduction:**

Pulmonary tuberculosis is considered one of the major public health issues in Peru, thus un derstanding its real condition from different points of view will allow choosing the most appropriate ther apeutic approach and patient care.

**Objective:**

To determine the relationship between social support and self-care in patients in the Lima Este Hospital, Peru.

**Materials and Methods:**

A quantitative correlational cross-sectional study was conducted with 114 patients participating in the NTCP program. Non-probability purposive sampling was built with 100 patients for which inclusion and exclusion criteria were applied. The MOS Social Support Survey and Self-Care Questionnaire were administered. Ethical considerations were considered for data collection. Data was processed using SPSS-24 software and later analyzed using descriptive statistics with frequencies and percentages. The chi-squared test was used for inferential anal ysis.

**Results:**

Sex, age, educational level and marital status had no relationship (p>0.05) with self-care in TB patients. Inappropriate self-care was found in 69.2% of patients receiving MDR-TB treatment. 100% of patients receiving drug-susceptible TB treatment showed appropriate self-care with a significant relation ship (p-value of 0.000). 83.3% of patients who perceived appropriate support showed appropriate self care, 76.9% of patients who perceived poor support rated their self-care as inappropriate with a significant relationship (p-value of 0.000). Similar findings were found for the social support dimension with a p-value of <0.05.

**Conclusions:**

Patients were characterized as young, male and single under drug-susceptible TB treatment. A significant relationship was found between appropriate social support and appropriate self care. A schedule for drug-susceptible TB treatment is related to inappropriate self-care.

## Resumo

**Introdujo:**

Um dos problemas de saúde pública no Peru é a tuberculose pulmonar, e compreender a realidade a partir de diferentes perspectivas permitirá uma abordagem adequada a terapia e cuidados para o indivíduo**.**

**Objetivo:**

Determinar a relacáo entre apoio social e autocuidado dos pacientes em um hospital em Lima - Este, Peru.

**Materiais e Métodos:**

Um estudo quantitativo, correlacional e transver sal. A populado era de 114 pacientes pertencentes ao Programa Nacional de Controle da Tuberculose (PNCT). A amostragem náo-probabilística por intenso foi utilizada, aplicando os critérios de inclusáo e exclusáo, a amostra consistiu em 100 pacientes. Foram aplicados a Escala de Apoio Social MOS e o Teste de Autocuidado. As considerares éticas foram levadas em conta na recolha de dados. Os dados foram processados no software SPSS-24, e analisados com estatísticas descritivas utilizando frequencias e percentagens. O qui-quadrado foi usado para análise inferencial.

**Resultados:**

Sexo, idade, nível de escolaridade e estado civil náo tiveram relacáo com o autocuidado de pacientes com TB com valor de p >0,05. 69,2% dos pacientes que receberam o regime de tratamento da TB MDR tinham um autocuidado inadequado. 100% dos pacientes que receberam tratamento para a tuberculose sensível mostraram um autocuidado adequado, com uma relacáo significativa com um valor p de 0,000. 83,3% dos pacientes que perceberam apoio adequado tinham autocuidado adequado, 76,9% que perceberam apoio defi ciente classificaram seu autocuidado como inadequado com uma relacáo significativa com um valor p de 0,000. Resultados semelhantes foram observados para as dimensoes de apoio social com um valor p<0,05.

**Conclusáo:**

Os pacientes caracterizam-se por serem jovens, homens, solteiros e receberem tra- tamento para a tuberculose sensível. Existe uma relacáo significativa entre o apoio social adequado e o autocuidado adequado. Um regime de tratamento para a TB resistente está associado a um autocuidado inadequado.

## Introducción

La tuberculosis es una enfermedad infectocontagiosa que constituye una de las principales causas de morbilidad. Continúa siendo un grave problema de salud pública en la región de las Américas y en el mundo, configurando como una de las causas de fallecimiento, aún sobre poniéndose al VIH^1^. La TB es causada por el bacilo *Mycobacterium tuberculosis*, que se propaga cuando los seres humanos infectados despiden o expulsan bacterias al espacio, al toser, escupir, estornudar o hablar. Ante esta reacción el sistema inmunológico se ve afectado por el microor ganismo, y se desencadena un grupo de signos y síntomas. Aproximadamente una cuarta parte de la población mundial está infectada. Uno de cada 3 habitantes del planeta está infectado con el bacilo de la TB y lo más dramático en pleno siglo XXI, es que cada año nueve millones de personas adquieren esta enfermedad^2^.

La tasa de incidencia más alta se registró en Haití, Perú y Bolivia con 176, 123 y 108 casos por cada 100.000 habitantes en 2018, respectivamente. La región LA y Caribe sigue enfrentándose a grandes desafíos en el control de la TB, entre ellos la prestación de servicios para las personas más vulnerables^3^. La OMS estimó que en el 2019 hubo 290 000 casos nuevos y recaídas de TB en la Región de las Américas. La cifra representa un aumento con respecto al 2018, cuando se estimaron 282 000 casos, y corresponde a 3% de la carga mundial de 9,9 millones de casos. En el 2019, se estimó que 10% de los pacientes de las Américas tenían coinfección TB/VIH y 3,7% pre sentaban TBs resistente a la rifampicina o multirresistente (TB-RR/MDR)^4^. Brasil tiene el 33,1% con 96000 casos estimados y 45,5 de tasa estimada; Perú 13,4%, con 39000 casos estimados y 120 de tasa estimada; México 10,3%, con 30000 casos estimados y 23,5 de tasa estimada y Co lombia 6,6%, con 19000 casos estimados y 35,8 de tasa estimada^5^.

En el caso de Perú, según el informe de la Organización Panamericana de la Salud (OPS), es uno de los países con mayor número de casos estimados de Tuberculosis en las Américas^6^. A nivel de la región de las Américas, Perú ocupa el tercer lugar entre los países con más alta tasa de incidencia, luego de Haití y Bolivia; y el primer lugar en reportar casos de TB resistente a medi camentos, reportando el 30% de los casos de TB MDR y el 50% de los casos de TB Extensamente Resistente (TB XDR) notificados. Las tasas notificadas de morbilidad total e incidencia por cada 100,000 habitantes, en los últimos 10 años muestran una tendencia sostenida en la disminución entre 1 a 3% anual. En el año 2016 se reportó una tasa de morbilidad de 98,7; una tasa de inci dencia total de 86,4; tasa de incidencia TB Pulmonar Frotis Positivo (TBP FP) de 53.2; mientras que la tasa de mortalidad evidencia una tendencia al incremento de 3.5 a 4 defunciones por cada 100 000 habitantes en los últimos 5años^7^.

La mayor morbilidad e incidencia de TB se da en la periferia de las ciudades capitales de departamentos del litoral del país, donde se asienta el 52% de la población nacional^8^. La zona donde se desarrolla el presente estudio, distrito de Ate, Hospital de Huaycán, es considerado como el segundo distrito sanitario con mayor cantidad de pacientes con TB a nivel de Lima, donde las tasas encontradas para la micro red fueron entre 2,6 y 4,9 veces superiores a las tasas descritas para el país^9^.

Un paciente con TB se encuentra afectado física y emocionalmente, ya que confronta una situa ción difícil en la parte económica, emocional y social, experimentando sentimientos de debilidad y aislamiento social^10^. Un aislamiento involuntario, ocasionado por la misma sociedad o su entorno más cercano, donde el paciente al no ser comprendido, se percibe como una persona contagiosa y con ello viene el deterioro emocional. Ante la falta de apoyo social el autoconcepto de las personas se ve alterado y casi siempre se encuentran decaídos, hasta pueden cortar toda comunicación, se niegan a los amigos, aduciendo que están de viaje, no salen de casa, y otros pueden renunciar a sus actividades laborales^11^**.** A pesar de no haberse reportado casos de suicidio derivados de un cuadro de TB, pero si hay estudios que reportan que los pacientes con TB que no cuentan con apoyo social presentan ideación suicida^12^^,^^13^.

Para el tratamiento de la TB se necesita un tiempo prolongado, es así que una de las causas del fracaso terapéutico es el abandono al tratamiento, por falta de voluntad y/o deterioro del apoyo familiar^14^. En el contexto del tratamiento y recuperación del paciente con TB es vital el apoyo familiar, se refiere al apoyo incondicional de la familia y comprende la satisfacción de las nece sidades de las situaciones cotidianas. Se caracteriza por la integración, participación, fuentes de apoyo recibido y proporcionado en el aspecto emocional, instrumental, relaciones sociales y afectivas, que van influir de manera positiva en la salud física y mental del paciente^15^. Las necesidades de apoyo social aumentan cuando hay un miembro enfermo.

El apoyo social se define como la información que lleva a las personas a creer que de algún modo son cuidados, amados, estimados y son miembros de una malla de obligaciones mutuas o solidarias. Es crucial porque permite el acompañamiento durante el proceso que enfrenta a la enfermedad, evitando pensamientos negativos e incentivando la autoestima^16^. Conceptuado en términos cognitivos como la percepción de que se es amado y estimado por los demás, su pone una percepción que promueve la salud, alivia el impacto de las enfermedades crónicas e influye beneficamente en la salud integral^17^.

El apoyo social percibido tiene un efecto positivo en el bienestar físico y emocional; esto in volucra el enfrentamiento adecuado a nuevas situaciones de manera proactiva el aislamiento social^18^. Es importante, pues proporciona ventajas en la mejoría del paciente^19^. Es por ello, que es muy valioso educar al paciente, a la familia y por extensión a la sociedad a fin de generar la unidad familiar y social en el control de la TB. La familia toma la participación de manera activa y de forma trascendental para favorecer y fortalecer el cumplimiento del tratamiento, no solo farmacológico sino también los cambios en sus hábitos y costumbres de vida^20^.

Para el logro del control de la TB es necesario que los pacientes desarrollen un autocuidado eficaz. El autocuidado, es considerado como un conjunto de acciones encaminados a atenderse a sí mismo. Es la capacidad de la persona en estado saludable, de enfermedad o de discapaci dad, para comprometerse con actividades de promoción de la salud física, mental y emocional, mantener la vida y prevenir las complicaciones ya sea por cuenta propia o en colaboración con su familia y los servicios de salud^21^. Siendo que la TB se acompaña de un debilitamiento general, con ausencia de la energía y capacidad física requerida para la realización de las actividades, el autocuidado es importante en la recuperación de la salud^22^. El autocuidado es un concepto o un término interpuesto por Dorothea E Orem en 1969 y es una actividad aprendida y comprendida por los individuos^23^.

Los estilos de vida son un conjunto de hábitos que tiene la persona. Si un paciente tiene un esti lo de vida saludable, se asocia a un autocuidado adecuado, mientras mejor sea el estilo de vida de un paciente, éste tendrá un mejor autocuidado. Respecto al autocuidado que debe tener todo paciente con TB, está claro que los estilos de vida saludables pueden prevenir la aparición de complicaciones y lograr la recuperación de su salud^24^.

Muchas infecciones presentan un curso prolongado y ocasionan secuelas de diferente índole. Según datos de la Organización Mundial de la Salud (OMS), entre las cinco primeras causas de mortalidad infecciosa a escala mundial se hallan tres enfermedades que cumplen esta condi ción y una de ellas es la TB^25^. Dentro del equipo de salud que provee atención, está el profesio nal de enfermería quien otorga los cuidados necesarios y cubre las necesidades del paciente. La profesión de enfermería en su parte filosófica contribuye a que la persona obtenga el nivel adecuado de calidad de vida.

La enfermera desempeña un rol decisivo en los programas de control y se considera un socio trascendental en la lucha contra la TB. El cuidado de enfermería como toda actividad humana tiene una dimensión ética y moral que se fundamenta en principios y valores sociales humanís ticos, es por ello que es necesidad y primordial otorgar cuidado en forma integral y de calidad para replantear la convivencia del individuo en el seno familiar y social^26^.

El profesional de enfermería que atiende en el área de servicio de TB tiene alta relación con los pacientes durante todo el proceso de su tratamiento y recuperación, es por ello, que cuenta con la mejor oportunidad de dialogar ampliamente con los pacientes sobre la enfermedad que sobrelleva; de esta manera se contribuirá a fortalecer la calidad de vida de los pacientes^16^.

El apoyo social en enfermería es necesario para los pacientes con TB porque crea una sensación de seguridad, atención, confort y comodidad aceptando su estado de salud y no aislándolos, su papel es ayudar a eliminar las actitudes negativas, como los sentimientos de inferioridad^27^. Por lo mencionado, enfermería ayuda a alcanzar una sensación de comodidad, confianza, entusias mo por el tratamiento y eliminación de pensamientos negativos.

El rol del profesional de enfermería en el control de infecciones muestra tres etapas:

• Etapa de identificación, se refiere al diagnóstico de los momentos y tareas de riesgo de pro pagación de las infecciones.
• Etapa de transición, caracterizado por un trabajo interactivo con el equipo de salud, se informa y establece una comunicación adecuada y clara, para implementar normas y programas de control y educación, en los que también participa en la obtención de tasas de infecciones.
• Etapa de confirmación en la que implementa estrategias de mitigación a los factores de riesgo. De manera permanente mostrará sus habilidades de epidemióloga, educadora, investigadora, comunicadora y participante de grupos para proyectos^28^.

Promover la adherencia al tratamiento de la TB implica alentar el autocuidado y empodera- miento del paciente de forma activa para su salud. El personal de enfermería juega un pa pel fundamental en el control y cumplimiento al tratamiento de los pacientes. La conexión se puede mejorar a través de una atención enfocada en el paciente, capacitándolos a través del asesoramiento y apoyo, manteniendo un enfoque basado en los derechos. La intervención de enfermería se destaca en el seguimiento al tratamiento, visita domiciliaria, educación, control de signos vitales y apoyo emocional, si el paciente abandona el tratamiento y no se hace una buena intervención de enfermería, el paciente con TB se hará resistente al tratamiento^29^.

El presente estudio se realizó en el Hospital Huaycán, zona poblada mayormente por personas provenientes del interior del país, con diferentes costumbres, vivencias, educación y estilos de vida. El objetivo fue determinar la relación que existe entre el apoyo social y el autocuidado de los usuarios del Programa Nacional de Control de Tuberculosis (PNCT) en el Hospital Huaycán.

## Materiales y Métodos

Estudio observacional con enfoque cuantitativo, de tipo correlacional y de acuerdo al momento del levantamiento de los datos es de corte transversal. El trabajo de investigación se realizó entre los meses de abril a diciembre del año 2020 con los pacientes que tienen el diagnóstico de TB pulmonar atendidos en el (PNCT) del Hospital Huaycán, distrito de Ate, Lima, Perú.

La población fue de 114 pacientes ambulatorios pertenecientes al PNCT (estadísticas del Hospital Huaycán). Se utilizó el muestreo no probabilístico por intención y se obtuvo la muestra constituida por 100 pacientes aplicando los criterios de inclusión y exclusión. Fueron incluidos los pacientes con TBP sensible a los medicamentos y TBP - MDR multidrogorresistentes. Asimismo, se excluyeron a pacientes que, a pesar de estar inscritos en el programa, no se les ubicó en sus viviendas y aquellos que no aceptaron participar del estudio.

El instrumento fue el Test de Apoyo social percibido, desarrollado por Sherboune y Stewarten (1991), en pacientes del Medical Outcomes Study (MOS). Cuenta con 20 preguntas de evalua ción. La primera pregunta valora el apoyo social cuantitativo. Ello hace posible identificar dos dimensiones de la red social: su composición en los aspectos familiar, extrafamiliar o mixta y su tamaño, que hace referencia al número de personas que componen la red social. Las siguientes 19 corresponden a 4 dimensiones: “Apoyo emocional” referido a las demostraciones de amor, cariño, estima y empatía. Disponibilidad de esas personas de las que puede recibir afecto. Per cepción de cómo se siente amado o admirado (ítems: 3, 4, 8, 9, 13, 16, 17 y 19), “Apoyo instru mental” se refiere a la ayuda material o asistencial, apoyo económico, colaboración para tareas domésticas (ítems 2, 5, 15 y 12), “Interacción social positiva”, es la disponibilidad para reunirse, divertirse o pasarlo bien con amigos o familia (ítems: 7, 11, 14 y 18 ) y “Apoyo afectivo” es la po sibilidad de recibir expresiones o demostraciones reales de amor, cariño o empatía (ítems: 6, 10 y 20 ). El presente estudio utilizó la versión en español de Revilla y Bailón (2014)^30^. Las opciones de respuesta están dadas a través de una escala likert de 1 (Nunca) a 5 (Siempre). La forma como se evaluó su equivalencia semántica del cuestionario se presenta a continuación: La puntuación final del apoyo social se agrupó en escaso (15-55 puntos) y adecuado (55-95 puntos)^31^^,^^32^.

Para el presente estudio las autoras realizaron la identificación sobre la validez de contenido por juicio de expertos donde se obtuvo un puntaje de 1.00. Asimismo, se realizó un estudio piloto donde se encontró la confiabilidad de 0.96 identificado con el coeficiente Alpha de Cronbach.

La variable autocuidado fue medida con un instrumento que se adaptó para pacientes con TB del test PEPS-I (Nola Pender)^33^, para lo cual se tomó en cuenta los aspectos teóricos del cons- tructo. Para hallar la validez del instrumento se sometió al análisis de expertos en la materia, contando con la participación de médicos (3) y enfermeros con grado de maestría y doctorado (7) y con amplia experiencia en el tema. Se obtuvo un valor de V de Aiken de 1.00, demostrando excelente validez. Pasó por dos rondas, ya que se hicieron ajustes a los ítems. Con los ajustes sugeridos por los expertos se realizó un estudio piloto donde se encontró la confiabilidad de 0.95, identificado con el coeficiente Alpha de Cronbach. Finalmente el instrumento quedó con formado con 25 preguntas divididas en 4 dimensiones: “Nutrición/Hidratación” (ítems: 1,2,3,4,5, 6,7,8), “Descanso y sueño” (ítems 9,10,11, 12,13), “Cuidado preventivo” (ítems 14,15,16,17,18,19) y Cumplimiento esquema de tratamiento” (ítems 20,21,22,23,24,25). Se mide en escala de Lic- kert que va desde nunca (1) hasta siempre (5). La puntuación final de autocuidado lo clasifica en adecuado (75-125 puntos) e inadecuado (25-75 puntos)^34^.

El levantamiento de datos se realizó tomando en cuenta la ética en investigación. Se solicitó au torización al Departamento de Docencia e Investigación del hospital de Huaycán, el cual, luego de un proceso de analisis otorgo la autorización. Para la aplicación de los instrumentos se cum plieron las normas de ética con total respeto a la dignidad de la persona y la confidencialidad y secreto de los datos recogidos. Se explico a los pacientes, los mismos que fueron ambulatorios, la naturaleza y objetivos de estudio. Finalmente, se solicito su consentimiento informado. La recolección de datos se realizó tomando todos los cuidados del protocolo del cuidado para COVID-19.

Para el análisis de datos, se utilizó el paquete estadístico SPSS v.24, en el cual se realizó la lim pieza de datos y se halló la confiabilidad de los instrumentos. Por otro lado, las variables cate góricas se presentaron en tablas de frecuencia simple. Para el análisis bivariado, se obtuvieron tablas de contingencia y se aplicó la prubea de Chi- cuadrado para variables nominales, debido a que las variables no seguían una distribución normal con un p valor < 0,05. La base de datos fue almacenada en Mendeley Data^35^

## Resultados

De 100 pacientes con TBP encuestados, el 64% fueron hombres y el 36% mujeres; pertenecien do el 72% al grupo etario de joven (18-19 años) y el 28% de adultos (30-59 años). Asimismo, el 29% presentó un grado de instrucción de técnico y el 61% manifestó estar soltero. En relación al esquema de tratamiento, el 64% de los pacientes recibía tratamiento para TB sensible y el 36% para TB resistente (Tabla 1).

**Tabla 1:** Características generales de los pacientes.

	n=100	%
Edad		
Joven (18-29 años)	72	72,0
Adulto (30-59 años)	28	28,0
Sexo		
Masculino	64	64,0
Femenino	36	36,0
Grado de instrucción		
Primaria	9	9,0
Secundaria	46	46,0
Técnico	29	29,0
Superior	16	16,0
Estado civil		
Soltero	61	61,0
Casado	12	12,0
Conviviente	24	24,0
Divorciado	3	3,0
Esquema de tratamiento		
TB sensible	64	64,0
TB resistente	36	36,0

**Figura 1:**
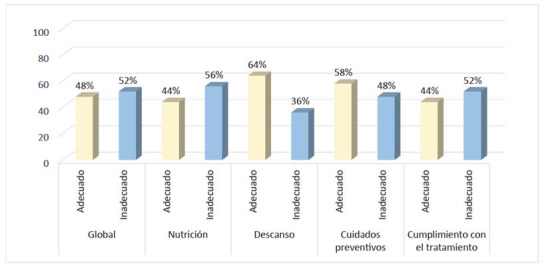
Autocuidado de los pacientes con TB

Al análisis descriptivo de las variables, se encontró que, el 52% de los pacientes presentó un autocuidado inadecuado y el 48% adecuado. De igual manera, en las dimensiones nutrición y cumplimiento con el tratamiento, se obtuvieron calificaciones inadecuadas en el 56% y 52% de los encuestados, respectivamente. Por el contrario, se obtuvo un autocuidado adecuado, en las dimensiones: descanso con un 64% y cuidados preventivos con un 58% (Figura 1).

**Figura 2:**
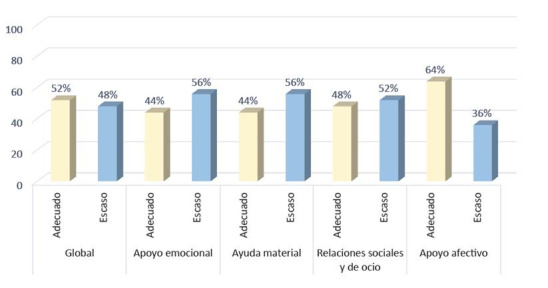
Apoyo social percibido por los pacientes.

Seguidamente, para la variable apoyo social, el 52% de los pacientes lo percibió como adecuado y el 48% como escaso. Del mismo modo, la dimensión apoyo afectivo fue valorada como adecuada en el 64% de los encuestados. Sin embargo, las dimensiones apoyo emocional, ayuda material y relaciones sociales y de ocio, obtuvieron una calificación de escasa, en el 56%, 56% y 52% de los pacientes, respectivamente (Figura 2).

Al análisis bivariado de las variables, se encontró que el sexo, la edad, el grado de instrucción y el estado civil, no tienen relación significativa con el autocuidado de los pacientes con TBP con p-valor >0,05 (Tabla 2). Por otra parte, se halló que el 69,2% de los pacientes que recibía esque ma de tratamiento para TB MDR presentaron un autocuidado inadecuado, mientras que, en el 100% de los pacientes que recibía tratamiento para TB sensible presentaron un autocuidado adecuado. Asimismo, esta relación fue significativa con un p-valor de 0,000 (Tabla 2). Respecto al apoyo social, se encontró que el 83,3% de los pacientes que percibieron un apoyo adecuado presentaron un autocuidado adecuado, en tanto que, 76,9% que percibió un apoyo escaso, calificó su autocuidado como inadecuado. A su vez, esta relación fue significativa con un p-valor de 0,000. Resultados similares se observan para las dimensiones del apoyo social con un p-valor <0,05 (Tabla 2).

**Tabla 2:** Asociación de las características generales y el apoyo social según el autocuidado

Variables	Autocuidado				p-valor
	Adecuado		Inadecuado		
	n	%	n	%	
Sexo					
Masculino	32	66,7	32	61,5	0,594
Femenino	16	33,3	20	38,5	
Edad					
Joven	35	72,9	37	71,2	0,844
Adulto	13	27,1	15	28,8	
Grado de instrucción					
Primaria/Secundaria	29	60,4	26	50	0,296
Superior/Técnico	19	39,6	26	50	
Estado civil					
Soltero/divorciado	35	72,9	29	55,8	0,074
Casado/conviviente	13	27,1	23	44,2	
Esquema detratamiento					
Sensible	48	100	16	30,8	0,000
MDR	0	0	36	69,2	
Apoyo social					
Escaso	8	16,7	40	76,9	0,000
Adecuado	40	83,3	12	23,1	
Apoyo Emocional					
Escaso	4	8,3	52	100	0,000
Adecuado	44	91,7	0	0	
Apoyo instrumenta l					
Escaso	8	16,7	48	92,3	0,000
Adecuado	40	83,3	4	7,7	
Interacción social positiva					
Escaso	8	16,7	44	84,6	0,000
Adecuado	40	83,3	8	15,4	
Apoyo afectivo					
Escaso	0	0	36	69,2	0,000
Adecuado	48	100	16	30,8	

## Discusión

En el presente estudio de investigación los pacientes con TB presentaron un mayor porcentaje el sexo masculino (64%). Con respecto al estado civil la mayor parte de los pacientes fueron solteros y con edad entre 18 y 25 años; con grado de instrucción secundaria (46%). En compa ración con el estudio realizado Chengliang en China en la provincia de Zhejiang, en su investigación entre los 212 participantes, había más pacientes varones con TB-MDR (69,81%).

El nivel de educación fue bajo con 164 (77,36%) pacientes que solo habían asistido a la escuela secundaria o primaria o no habían recibido ninguna educación. Los grupos de edad de 40 a 59 años representaron el 43,87%, respectivamente^36^. Las personas del genero masculino son los mas propensos a tener un comportamiento con un mayor riesgo de contraer TB, lo que incluye fumar, consumir alcohol y drogas^37^. Así mismo, los factores por la cual las personas son más propensas a contraer TB es ser económicamente desfavorecido, actividades inadecuadas para una vivienda saludable.

En cuanto al objetivo general del presente estudio fue demostrar si existe relación entre el apo yo social percibido y el autocuidado y se encontró que si existe relación significativa entre un adecuado apoyo social y un adecuado autocuidado. De igual manera en un estudio realizado por Sukartini en Indonesia, el apoyo familiar tiene relación con la conducta de autocuidado, de donde se deduce que la familia es la fuente más importante de apoyo social puesto que, influye en el comportamiento de autocuidado y puede reducir o mejorar el estado de salud y el bienestar^38^.

En el presente estudio se hallo relación apoyo social y el autocuidado, dado que una de las di mensiones es nutrición, consideramos que existe correlación. De manera similar en un estudio realizado en España por García et al, se observó la correlación entre el apoyo social para los hábitos alimentarios y la percepción de sufrir presión externa para cambiar (r=0,26; p=0,001)^39^.

Mientras mas alto es el nivel de apoyo social, mejor será el estado de nutrición de los pacientes puesto que este es factor en la prevención y control de la TB, por tanto un buen autocuidado.

De la misma manera una dimensión de la variable autocuidado es el “descanso y sueño” en el presente estudio se obtuvo una relación con la variable apoyo social. En el estudio realizado en la Micro Red de Concepción de Perú, encontraron que existe relación directa y significativa entre los conocimientos y actitudes para descanso y sueño sobre medidas preventivas en con tactos de pacientes con TBP de (r=1.000;p=0.000<0.05). Para la realización de las actividades cotidianas y la capacidad de concentración es necesario emplear un buen descanso y tener un sueño completo ya que es esencial para la salud y básica para tener una excelente calidad de vida^40^.

La TB al ser una enfermedad infectocontagiosa requiere de cuidados adecuados las medidas que deben adoptar como práctica cotidiana los pacientes con esta enfermedad ayudará a concientizar de tal manera que pueda valorar su bienestar físico y emocional^41^. Además, no solo contribuye con la prevención sobre si mismo sino también en forma indirecta estará aliviando la presión sobre el sistema sanitario del país. Es por eso que, es muy importante que cada persona al tener buenos hábitos de autocuidado personal estará en condiciones de tomar mejores deci siones acerca de su salud, el mismo que garantiza la responsabilidad sobre su propio bienestar y de la familia que lo rodea. También, adoptar tales decisiones de prevención con criterio rigu roso, estará apoyando a la sociedad ya que el paciente puede sentirse emocionalmente bien consigo mismo y con su vida. De ahí que el apoyo social es fundamental para el tratamiento preventivo, curativo y de rehabilitación de los mismos^42^.

En el estudio realizado por Lawrente, respecto al conocimiento sobre las medidas preventivas, identificó que 67.1% de pacientes calificaron su conocimiento acerca de la prevención de la TB como adecuado y los 32.9%, lo calificaron como inadecuado^43^. Por otro lado Mejia en su investigación Factores asociados a los conocimientos sobre TBP las personas en el pre test ob tuvieron un 7% de conocimientos correctos en las medidas de prevención para la TB y luego de la intervención educativa obtuvieron el 100% de conocimientos correctos^44^. Esto evidencia que mantener capacitaciones activas en los centros de salud por parte del personal de enfermería mejora el nivel de conocimiento tanto del paciente como del familiar.

La problemática social de esta enfermedad actualmente se centra en el riesgo de incumpli miento terapéutico y su posible influencia negativa tanto en la salud del paciente como en el control de la enfermedad desde el punto de vista de la salud pública. El apoyo social influye sobre las decisiones que tomaría con respecto al cumplimiento del tratamiento de la enferme- dad^44^. El personal de Enfermería se encuentra en mayor contacto con el paciente durante todo el proceso de su tratamiento y por ello, contará con la mejor oportunidad de conversar con él sobre su enfermedad, reacciones adversas, controles mensuales, y el seguimiento luego del alta^45^. El apoyo social influye de manera directa en el paciente, ya que se sabe que la TBP daña su autoestima generando sentimientos de culpa, depresión, resentimiento y vergüenza, ya que son factores que van a influir negativamente en el adecuado cumplimiento del tratamiento.

En un estudio realizado en Armenia se evidencia en sus resultados que de el 98%) informaron haber recibido paquetes sociales durante el tratamiento y 96% estaban satisfechos. La mayoría de los pacientes prefirieron incentivos monetarios (57,8%) en lugar de los kits de alimentos e higiene que se proporcionan actualmente. El éxito se asoció positivamente con la satisfacción con el apoyo social proporcionado (razón de posibilidades (OR) = 2,8, intervalo de confianza (IC) del 95%: 1,0; 7,6, P = 0,04)^46^. Las intervenciones de apoyo social tienen un gran potencial por ser métodos efectivos para mejorar los resultados del tratamiento para los pacientes con TB-DR^47^.

Cuando el paciente percibe el apoyo, ayuda y preocupación de la familia, se sentirá más con fiado y motivado a culminar con el tratamiento para poder recuperarse pronto y reinsertarse a su vida cotidiana, por otro lado, el apoyo social influye de manera directa en el paciente, ya que se sabe que la TBP daña su autoestima generando sentimientos de culpa, depresión, re sentimiento y vergüenza, ya que son factores que van a influir negativamente en el adecuado cumplimiento del tratamiento.

Los pacientes con TB, generalmente se encuentran en una etapa sensible debido a diversos cambios en su estilo de vida, por la presión de sí mismo como persona, de la familia y más que todo en el ámbito social, lo familiar puede ser tolerante, pero de parte de la sociedad muchas veces viene el rechazo, el paciente lo siete en los gestos, en la mirada o “murmuraciones” que lo percibe como rechazo por el peligro del contagio.

El presente estudio esta referido a los pacientes inscritos en el programa del hospital, razón por la que se tomó a esa población y se utilizó el muestreo no probabilistico censal, sin embargo en proximos estudios se puede tomar a los pacientes no inscritos en el programa del hospital, es decir a los que acuden a otros centros de atención y utilizando un muestreo probabilístico se puede extrapolar los resultados. Debido a que se utilizó la categorización dicotómica se ha uti lizado el Chi cuadrado, sin embargo a fin de mejorar la visibilidad de los resultados, se presenta en figuras.

## Conclusiones

El estudio reporta que los pacientes con TBP que se atienden en el Hospital de Huaycán se ca racterizan por ser jóvenes, varones, solteros y recibir tratamiento para TB sensible. Se demostró que existe relación significativa entre un adecuado apoyo social y un adecuado autocuidado. Por otro lado, un esquema de tratamiento para TB resistente se relaciona con un inadecuado autocuidado.

El estudio es importante debido al rol que cumplen los enfermeros, los resultados pueden ser vir de base para generar propuestas que se traduzcan en estrategias o políticas. Consideramos importante incorporar otras metodologías como la investigación cualitativa y la investigación mixta, a fin de tener un panorama más amplio. De la misma manera estudios multidisciplinarios a fin de generar consensos con un enfoque intercultural.
